# One Health Priorities: Advancing Veterinary Public Health in Latin America and the Caribbean

**DOI:** 10.3390/pathogens13080710

**Published:** 2024-08-21

**Authors:** Felipe Rocha, Alessandra Cristiane Sibim, Baldomero Molina-Flores, Wagner Antonio Chiba de Castro, Louise Bach Kmetiuk, Renato Vieira Alves, André Luis de Sousa dos Santos, Margarita Corrales Moreno, Álvaro A. Faccini-Martínez, Natalia Margarita Cediel, Alexander Welker Biondo, Ottorino Cosivi, Marco Antonio Natal Vigilato

**Affiliations:** 1 Pan American Center for Foot-and-Mouth Disease and Veterinary Public Health (PANAFTOSA/VPH), Pan American Health Organization (PAHO/WHO), Rio de Janeiro 25045-002, RJ, Brazil; rochafe@paho.org (F.R.); molinab@paho.org (B.M.-F.); cosivio@paho.org (O.C.); 2 Latin-American Institute of Technology, Infrastructure and Territory, Federal University for Latin American Integration (UNILA), Foz do Iguaçu 85870-650, PR, Brazil; 3 Latin-American Institute of Life and Nature Sciences, Federal University for Latin American Integration, Foz do Iguaçu 85870-650, PR, Brazil; wagner.castro@unila.edu.br; 4 Zoonosis Surveillance Unit, Curitiba 81265-320, PR, Brazil; 5 Servicio de Infectología, Hospital Militar Central, Facultad de Medicina, Bogotá 110231, Colombia; 6 Facultad de Medicina, Universidad Militar Nueva Granada, Bogotá 110110, Colombia; 7 Servicios y Asesorías en Infectología, Universidad Militar Nueva Granada, Bogotá 110111, Colombia; 8 School of Agricultural Sciences, De La Salle University, Bogotá 11001, Colombia; 9 Department of Veterinary Medicine, Federal University of Paraná, Curitiba 80035-050, PR, Brazil; abiondo@ufpr.br

**Keywords:** sustainability, One Health index, veterinary public health, public policy, climate change

## Abstract

One Health (OH) is an integrative approach to human, animal, and environmental health and can be used as a comprehensive indicator for comparative purposes. Although an OH index has been proposed for comparing cities, states, and countries, to date, no practical study has compared countries using this approach. Accordingly, this study aimed to assess OH initiatives using a survey with a veterinary public health focus. The questionnaire contained 104 quantitative questions and was sent to representatives of governmental institutions of 32 countries in the Americas. After exclusion criteria were considered, a total of 35 questionnaires from 17 countries were analyzed, with country names remaining undisclosed during the statistical analyses to protect potentially sensitive information. Principal component analysis (PCA) of health parameters in Latin America and the Caribbean (LAC) as a function of country perception (self-vector) showed that food safety was ranked higher than public policies (*p* = 0.009), and that both (*p* = 0.003) were ranked higher than institutional routines related to zoonosis programs. National policies in accordance with international standards, regulations, recommendations, and guidelines was considered the standout topic for public policy, with higher-ranking topics including standard. Meanwhile, challenging topics included tools, preparedness, governance, and research. Food safety showed both strengths and challenges in the coordination of its activities with other sectors. Food safety communication was scored as a strength, while foodborne diseases prevention was ranked as a challenge. Institutional routines for zoonosis maintained both strong and challenging topics in the execution and implementation of attributions and daily routine. Thus, the survey showed that topics such as access to and compliance with international guidelines and intercountry integration were ranked higher than in-country articulation, particularly among food safety, zoonoses, and environmental institutions.

## 1. Introduction

Despite its longstanding application before the term was coined, and its extensive study in recent years [1], the practical implementation of the One Health (OH) approach in governmental processes remains a challenge, as does surveying its initiatives and indicators, including in Latin America and the Caribbean (LAC) [2]. Several perception questionnaires have recently been proposed and applied, varying according to survey subject and geographic region. Such studies include neglected vector-borne infections [3] and foodborne zoonoses, antimicrobial resistance, and emerging microbiological hazards [4] in European countries; a zoonosis survey in the Americas [5] and health responses to global challenges in Colombia and other LAC countries [6]; OH assessments in Sub-Saharan African countries [7], in French-speaking countries [8], and in the French territory Guadeloupe [9]; and an OH index applied in a major metropolitan area of Brazil, city of Curitiba [10]. In addition, surveys on the OH approach have been proposed for zoonotic disease control at all levels [11], including OH initiatives in Asia [12], synergizing tools for OH operationalization [13], OH matrix surveillance [14], and the Global OH Index [15].

The Pan American Health Organization (PAHO) has been promoting a multisectoral approach to managing risks at the human–animal interface for several decades through its veterinary public health technical cooperation support. PAHO’s Inter-American Ministerial Meeting on Health and Agriculture (RIMSA), first established in 1968, most recently met in 2016 to discuss “One Health and the Sustainable Development Goals” [16]. Furthermore, in September 2021, the 59th Directing Council officially approved PAHO’s OH policy “One Health: A Comprehensive Approach for Addressing Health Threats at the Human-Animal-Environment Interface (Document CD59/9)”, prioritizing endemic diseases of zoonotic and vector-borne origin, emerging and re-emerging infectious diseases of zoonotic origin, antimicrobial resistance, and food safety [17]. Resolution CD59.R4, endorsed the policy and called for Member States to implement OH and for the Pan American Sanitary Bureau to provide the related technical cooperation support [18].

PAHO’s OH policy proposes the analysis and mapping of OH health interactions in specific national contexts, the establishment of OH governance, strengthening multidisciplinary and intersectoral aspects, emergency preparedness and response, digital technology and scientific tools, research, and capacity building. Following PAHO’s OH policy, the six strategic lines of action for the implementation of OH into national policies were included in the question.

Within the Pan American Sanitary Bureau there is the Pan American Center for Foot-and-Mouth Disease and Veterinary Public Health (PANAFTOSA/VPH-PAHO/WHO), a specialized PAHO Center responsible for veterinary public health that is providing technical cooperation to both public health and official veterinary services of countries in LAC [19]. In July 2022, in Rio de Janeiro, Brazil, PANAFTOSA/VPH-PAHO/WHO coordinated a two-day meeting with 15 participants from the public health and official veterinary services sectors from 10 LAC countries (Argentina, Belize, Bolivia, Brazil, Chile, Colombia, Cuba, Honduras, Mexico, and Uruguay) to discuss OH. The meeting sought to identify OH best practices and gaps and address the need to gather information on OH policy implementation for veterinary public health in LAC countries.

There are several definitions of OH [17,20]; however, for the scope of this paper, OH is defined as an integrative approach to human, animal, and environmental health [1]. Therefore, OH can be used as a comprehensive indicator for comparative purposes [10]. Although an OH index has been proposed for comparing cities, states, and countries [10,15,21,22,23], no practical study has been conducted to compare countries using such an approach. Accordingly, the present study aimed to develop, apply, and analyze perception questionnaires as a basis for the assessment of OH strengths and challenges in LAC countries.

## 2. Materials and Methods

### 2.1. Survey Questionnaire

Thirteen recently published studies using questionnaires to assess OH were obtained from the available literature (Table 1). A comprehensive questionnaire focusing on veterinary public health was developed and reviewed as part of the activities discussed in the meeting coordinated by PANAFTOSA/VPH-PAHO/WHO. The questionnaire was sent to both the official veterinary service and the public health sectors of 32 LAC countries from December 2022 to March 2023. Responses from 42 institutions across 26 countries were gathered and used for statistical analysis (Supplementary Table S1). The questionnaire was available in three languages (Spanish, Portuguese, and English), having been discussed, reviewed, pre-tested, and unanimously approved by 15 institutions from 10 LAC countries on 12 and 13 July 2022 as part of the meeting organized by PANAFTOSA/VPH-PAHO/WHO. All institutions listed in the PAHO database related to human, animal, and environmental health–including national ministries of health, ministries of livestock and agriculture, and ministries of the environment–were contacted, and they received the online questionnaires through the PAHO channel. Professionals voluntarily answered the online questionnaire in their chosen language (Spanish, Portuguese, or English), and there was a common database for gathering and analyzing results.

### 2.2. Data Analysis

#### 2.2.1. Questionnaire Selection

Questionnaire outcome data based on the perceptions of OH by different delegates representing their respective countries were thoroughly examined. A total of 104 quantitative questions (rated on a 5-point Likert scale) were used, with open-ended questions excluded from the analysis. Questionnaires with missing answers were also excluded. When more than one answer was sent by a given country (i.e., more than one professional answered the questionnaire), the final score was defined as the average score.

#### 2.2.2. Questionnaire Analysis

Questions regarding veterinary public health parameters were organized into three relevant thematic groups: (1) food safety–examining the perceived importance of OH in food safety in a given country; (2) public policies–exploring the perceived role of government in subsidizing OH in different areas, such as interaction, governance, internationalization, promptness, tools, and research; and (3) institutional routines related to zoonosis programs, searching for perceived role of OH in the daily work of the relevant professionals. A list of the 104 questions used for the database construction, and their allocation into thematic groups, is provided in Table 2.

Differences between the health parameters of OH in LAC countries were assessed using permutation multivariate analysis of variance (PERMANOVA) [24] and by searching for paired comparisons [25]. A *p*-value of 0.05 or less was considered significant. Strengths (positive aspects) and challenges (negative aspects) were assessed using a principal component analysis (PCA) as a function of country perceptions (autovectors). All statistical analyses were performed using the statistical software package R, version 4.4.1 [26]. The five highest-scoring questions (strengths) and five lowest-scoring questions (challenges) per thematic group were chosen, totaling 30 questions.

## 3. Results

**Figure 1:**
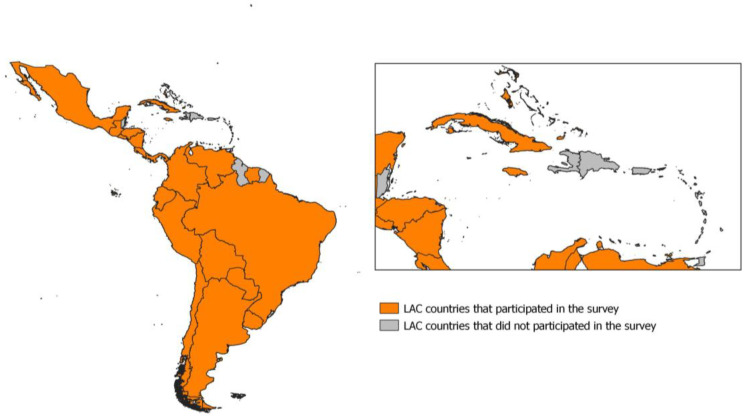
Countries participating in the One Health perception questionnaire.

The survey questionnaire was answered by 54 professionals from 26 LAC countries: Anguilla, Argentina, Aruba, the Bahamas, Bermuda, Bolivia, Brazil, the British Virgin Islands, Chile, Colombia, Costa Rica, Cuba, Ecuador, El Salvador, Guatemala, Honduras, Jamaica, México, Nicaragua, Panamá, Paraguay, Perú, Suriname, the Turks and Caicos Islands, Uruguay, and Venezuela. Once the exclusion criteria were applied, a total of 35 questionnaires from 17 countries were analyzed (Figure 1). Sensitive data regarding professionals, institutions, and countries in the survey questionnaires were not disclosed in statistical analysis or data presentation. The survey respondents were professionals in charge of zoonosis and food safety programs at their countries. In other words, they were decision makers at the national level who understood the situation in their countries. Despite the apparently small sample size, the value of the answers is considered of great significance.

**Figure 2:**
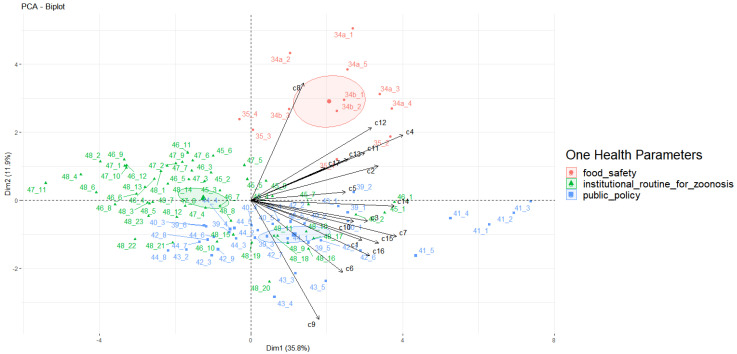
Graphic of principal component analysis showing the One Health perceptions of Latin American and Caribbean countries (17 self-vectors), with a focus on veterinary public health, based on the applied questionnaire (Supplementary Table S1). Colors indicate the veterinary public health parameters and ellipses indicate the confidence intervals (food safety in red, institutional routines for zoonosis in green, and public policy in blue).

PERMANOVA indicated significant differences between the OH thematic groups or parameters (Table 3). The PCA of health parameters in LAC as a function of countries’ perceptions (self-vectors) showed that food safety and public policies were evaluated better than institutional routines for zoonosis (Figure 2).

The questions with the highest and lowest scores for each health parameter are presented in Table 4.

## 4. Discussion

The present study has shown statistically significant OH strengths and challenges in LAC countries based on high- and low-scored answers from an official survey, conducted among national governmental institutions, that addresses public policies, food safety, and institutional routines related to zoonosis programs.

Several LAC countries have established OH public policies that incorporate health, social, and economic determinants and have developed health systems that integrate multisectoral interventions [27]. Thus, the data herein should accurately mirror OH demands, as countries have previously shown high familiarity (92%) and collaboration (68%) baselines regarding OH, despite almost half of the participants (46%) referring to the establishment of such connections in the past five years [6]. This study also showed that food safety was ranked among the top three human, animal, and environmental health issues in LAC countries, along with antimicrobial resistance and zoonoses [6].

Such engagement has also been found on other continents, as two-thirds of respondents (63%) from 34 European countries declared that they were taking part in OH initiatives [3]. A survey in sub-Saharan Africa obtained 57 responses from different African countries with 145 OH initiatives [7], and in a survey of French-speaking countries, the vast majority of respondents (98%) acknowledged OH as relevant, and most respondents (64%) had already implemented OH initiatives [8]. The One Health European Joint Programme (OHEJP) has also identified food safety and policy changes as priority topics, indicating that communication and dissemination are key components for the successful achievement of OH actions [4]. Similarly, the Asian Development Bank suggested an interconnected OH approach to development problems, proposing solutions through transdisciplinary communication, coordination, and collaboration [12]. In another survey in Australia the participants agreed that OH was essential for effective infectious disease prevention and control [28].

### 4.1. Public Policy

National policies in accordance with international standards was considered the standalone strength for public policy, meaning that the standardization of, understanding of, and trust in international standards and corresponding organizations obtained the highest topic scores in the present survey. These international guidelines comprise the legal framework of the International Health Regulations (IHR); the standards, codes of practice, and other recommendations of the Codex Alimentarius; the international standards provided by the World Organisation for Animal Health (WOAH); the Global Action Plan on Antimicrobial Resistance; and the prevention of the international spread of contaminated food and foodborne diseases by the International Food Safety Authorities Network (INFOSAN) [29].

The interpretation of such results may indicate satisfactory delivery of standards and guidelines by international organizations, including availability, language, promptness, and training. The COVID-19 pandemic showed the importance of international cooperation in responding to global health crises. International guidelines and public policies have also been shown to be important in preparing for addressing cross-border issues, as almost half of the guidelines and policies have been associated with zoonotic or food safety events [13].

Meanwhile, other topics related to tools, preparedness, research, and governance were identified as challenging issues that need to be addressed, obtaining the lowest scores in the survey. Including topics such as public health interventions and technology; strategy and technical activity; integration between governments and communities; and encouragement of OH research and development and scientific publication. Overall, organization structures and coordination and collaboration mechanisms are crucial for an effective OH approach and for monitoring strengths and weaknesses in a multisectoral surveillance system [14]. Other challenges are related to the formulation of the OH strategic roadmap and governance mechanisms, indicating the difficulty of internationalizing the implementation and application of the concept within national policies.

Thus, although the alignment between national and international policies was perceived as a strength, indicating favorable OH performance in LAC countries on the global scale, government–community integration should be addressed, as it remains a major challenge for institutional routines for zoonosis at the local level.

### 4.2. Food Safety

The highest and lowest scores for food safety in the present study indicated a positive cohesion between such topics. Topics related to the coordination with other sectors was simultaneously ranked as the strongest and the weakest characteristic for different food safety topics. Multisectoral coordination for preparedness and response to future emerging and re-emerging zoonotic diseases within a new OH framework has been demanded worldwide [30]. The strengths within topics related to the coordination between different technical areas include the surveillance of and authority coordination on foodborne diseases, along with contaminant issues and actions and communication between countries. Not surprisingly, stakeholders in governments, the food industry, and the research community should always work collectively to effectively address and communicate the safety of new food sources and production systems [31].

In contrast, the two lowest scoring topics in coordination were related to the management and disposal of pesticides and antimicrobials, which may serve as a warning about environmental contamination and pollution in LAC countries. Such harmful compounds may not be significantly removed in conventional wastewater processing and could be discharged into the environment, resulting in increased threats to human and animal health in anthropic and natural ecosystems [32].

The present study has also shown important positive advances in food safety understanding and concerns in LAC countries. Previously, disagreement on risk analysis in national food safety regulations has been found among professionals working in food safety in the academic, government, and private sectors [33]. Assessing 23 countries of the LAC in 2014, only 70/279 (25%) stakeholders were able to correctly identify key principles of food safety, indicating a systematic lack of understanding of risk analysis [33]. In addition, water quality and its use for consumption and food preparation should be reviewed by local authorities and governments to achieve the targets of the Sustainable Development Goals, based on an assessment of documents on the official websites of countries in LAC, the Food and Agriculture Organization of the United Nations, and the World Food Programme [34].

The concern about food safety is not restricted to LAC countries; it is also reportedly one of the most pressing issues in Asia and the Pacific, particularly due to the negative impact of the COVID-19 pandemic on food security [12]. In such a scenario, achieving nutrition security in Asia required an integrated, cross-sectoral approach to producing healthy and nutritious food in sustainable food chains and improving food safety and livestock biosecurity [12]. In LAC countries the COVID-19 pandemic emphasized the demands on local food systems, family farming, and agroecology movements, particularly when the demands were increased by public policy pressures [35]. Although countries made great efforts to align their legislation with international standards during the pandemic, internal coordination between authorities and countries remains to be fully established, particularly in surveillance and responses to food safety events.

Food safety is not a recent concern. A 17-year-long survey of bacterial foodborne disease outbreaks in 20 Latin American countries found that meat, dairy products, water, and vegetables in the 1990s and eggs, vegetables, grains, and beans in the 2000s were the leading sources of bacterial diseases [36]. In this study, the changes in food sources of infection were associated with a series of changes over time, including zoonosis control, food consumption habits, outbreaks of public health interest, and pathogen data availability. This study has indicated the impact of surveillance systems and their data gaps in Latin America with respect to satisfactorily identifying foodborne pathogen sources [36].

Within the food safety parameter, prevention of illness received low scores for the following topics: interactions between food control authorities and zoonosis, interactions between food control authorities and environmental authorities, and national approaches to creating an integrated surveillance network among countries. The challenges of the national integration and collaboration of food safety with institutional routines related to zoonosis programs remain to be fully addressed at the global scale through the OH approach. Unfortunately, ministries of the environment and other national and international environmental organizations have not yet been enlisted as part of the taskforce against zoonotic diseases, as reported in European countries [3]. Thus, as ministries of health and ministries of agriculture have been working alone, the enrolment of environmental health professionals in teams for the control and monitoring of such diseases should be considered a challenge for the practical establishment of the Global One Health.

### 4.3. Institutional Routines for Zoonosis

As expected, topics related to the execution and implementation of attributions and daily routine resulted as both strong and weak topics for institutional routines related to zoonosis programs. Strengths included geographic coverage, a focus on surveillance systems, identifying the hazards of particular disease groups, international collaboration and cooperation, and the capacity to provide information about epidemiological hazards. In LAC countries, established commissions and interprogram working groups are tasked with addressing emergencies and fostering intergovernmental cooperation through efficient communication channels [37,38]. The sense of urgency created by the COVID-19 pandemic notably expedited interprogram collaboration among diverse governmental entities, with collaboration focusing on combating emerging and re-emerging zoonotic diseases [39].

Several challenges persist, including feedback-loop mechanisms for corrective measures, the establishment of robust monitoring and evaluation plans, the sharing of statistical techniques, adopting common indicators for data analysis, and ensuring interoperability. Notably, the institutional routines for zoonosis parameter yielded poorer results than food safety and public policy in the PCA (see Figure 1), underscoring the difficulty of implementing the parameter within national activities. Despite local OH initiatives addressing public health concerns, countries lack a cohesive approach to organizing and structuring national OH mechanisms at higher levels [40].

To bolster institutional routines for zoonosis, countries must facilitate extensive discussions aimed at developing robust communication channels and fostering information exchange through integrated information systems. These systems should not only focus on reporting disease occurrences but also document the actions and measures implemented to control diseases. Emphasis should be placed on identifying how each country contributes to its ongoing health initiatives.

### 4.4. Final Considerations

In the present study, topics such as access to and compliance with international guidelines and intercountry integration were ranked higher than in-country articulation, particularly for interactions among food safety experts, zoonoses, and environmental institutions and professionals. Although international guidance may indicate preparedness for global issues, national (technical working group) and subnational (information sharing and joint reporting) levels have been equally crucial for successful OH approaches to event-based surveillance [11].

This study revealed a lack of appropriated animal health parameters at the private and governmental institution, city, state, ministerial, and country levels, and animal health indicators were limited to qualitative actions for the health and welfare of companion animals rather than livestock or wildlife species.

## 5. Conclusions

The present study has shown statistically significant OH strengths and challenges within national governmental institutions pertaining to public policy, food safety, and institutional routines related to zoonosis programs in LAC countries. Access to, and compliance with, international guidelines and intercountry integration were ranked higher than in-country articulation, particularly among food safety, zoonoses, and environmental institutions. Based on our results, the concept of OH demonstrates notable strengths within the food safety sector in LAC, this being particularly evident in its alignment with international standards, which provide valuable guidance for the development of national policies. However, despite this alignment of international standards, and their positive influences, the practical application of OH in daily activities and its effective implementation remain significant challenges for the national authorities that participated in this survey.

## Figures and Tables

**Table 1:** A review of 13 recently published studies with questionnaires for One Health assessment, obtained from the available international literature.

Applied Locations	Type of Survey	Reference
Pan-American countries	Prioritization of emerging and endemic zoonoses; countries’ prioritization criteria and methodologies	[5]
European countries	Joint actions on foodborne zoonoses, antimicrobial resistance, and emerging microbiological hazards	[4]
37 European countries and neighboring areas	Collection of information on the existence of OH collaboration and implementation of OH initiatives	[3]
Colombia and some Latin American countries	Assessment of a fragmented health organization in relation to an integrated health response to global challenges	[6]
Guadeloupe, French territory	OH operationalization in past and current collaborative initiatives and analysis of the OH framework	[9]
Sub-Saharan African countries	OH strengths, weaknesses, opportunities, and threats	[7]
French-speaking countries	OH applied to research, surveillance, and control of neglected tropical diseases	[8]
Major metropolitan area, Brazil	Comparative indicators of human, animal, and environmental health leading to an OH index	[10]
Asian countries	Experiences from previous studies as tools for capacity assessment and OH operationalization	[12]
Proposed	Examples and initiatives of national decision makers implementing OH	[13]
Proposed	Assessment of multisectoral collaboration by analysis of its organization, implementation, and functions	[14]
Proposed	Assessment of steps towards a global OH index	[15]
Proposed	Use of OH in zoonotic disease programs at local, national, regional, and international levels	[11]

OH = One Health.

**Table 2:** Relevant thematic groups of the One Health questionnaire with a veterinary public health focus and their correspondent question allocations.

Thematic Group on Veterinary Public Health Parameters	Question Number in the Survey (See Supplementary Table S1)
Food safety	34a_1, 34a_2, 34a_3, 34a_4, 34a_5, 34b_1, 34b_2, 34b_3, 35_1, 35_2, 35_3, and 35_4
Public policy	39_1, 39_2, 39_3, 39_4, 39_5, 39_6, 40_1, 40_2, 40_3, 40_4, 40_5, 41_1, 41_2, 41_3, 41_4, 41_5, 42_1, 42_2, 42_3, 42_4, 42_5, 42_6, 42_7, 42_8, 42_9, 43_1, 43_2, 43_3, 43_4, 43_5, 44_1, 44_2, 44_3, 44_4, 44_5, 44_6, 44_7, and 44_8
Institutional routines for zoonosis	45_1, 45_2, 45_3, 45_4, 45_5, 45_6, 45_7, 45_8, 46_1, 46_2, 46_3, 46_4, 46_5, 46_6, 46_7, 46_8, 46_9, 46_10, 46_11, 46_12, 47_1, 47_2, 47_3, 47_4, 47_5, 47_6, 47_7, 47_8, 47_9, 47_10, 47_11, 48_1, 48_2, 48_3, 48_4, 48_5, 48_6, 48_7, 48_8, 48_9, 48_10, 48_11, 48_12, 48_13, 48_14, 48_15, 48_16, 48_17, 48_18, 48_19, 48_20, 48_21, 48_22, and 48_23

**Table 3:** Results of the permutation multivariate analysis of variance that searched the paired comparisons among One Health parameters under the focus on veterinary public health.

Pairs of Parameters	Sums of Squares	F-Value	*p*-Value
Food safety vs. public policy	67.21559	4.676195	0.009 *
Food safety vs. institutional routines for zoonosis	142.5498	11.43743	0.003 *
Public policy vs. institutional routines for zoonosis	161.8979	12.2474	0.003 *

* Statistical significance of *p* ≤ 0.05.

**Table 4:** List of questions, organized by health parameter, which presented the highest and lowest scores attributed by countries, indicating the strengths of and challenges for One Health in Latin America and the Caribbean.

Thematic Group on Veterinary Public Health	Questions	Topic	Scores (Highest)	Scores (Lowest)
Public policy	41_3	WOAH and sanitary safety	4.31	
	41_2	Codex Alimentarius and food safety	4.23	
	41_1	IHR and public health	4.12	
	41_4	Global action plan and antimicrobial resistance	3.81	
	41_5	INFOSAN and food safety	3.72	
	43_2	Public health interventions and technology		2.35
	42_3	OH strategy and technical activity identification		2.32
	44_7	Encouragement of research and development		2.29
	40_3	OH integration between government and community		2.20
	44_8	Scientific journals’ encouragement of OH articles		2.12
Food safety	35_2	Surveillance of foodborne diseases	3.40	
	34a_4	Authority coordination on foodborne diseases	3.34	
	34a_3	Authority coordination on actions	3.34	
	34b_1	Better communication between countries	3.14	
	35_1	Control, monitoring, and reduction of contaminants	3.13	
	34a_1	Food control and zoonosis authorities		2.98
	34a_2	Food control and environmental authorities		2.84
	34b_3	Integrated surveillance between countries		2.77
	35_3	Management of agrochemicals and antimicrobials		2.62
	35_4	Disposal of agrochemicals and antimicrobials		2.50
Institutional routines for zoonosis	46_1	Geographic coverage	3.55	
	45_1	Common objective for surveillance systems	3.45	
	46_2	Hazards for the same disease groups	3.28	
	48_16	International collaboration and cooperation	3.21	
	48_17	Capacity to provide information about epidemiological hazards	3.02	
	46_9	Feedback loops (correction measures)		1.86
	46_8	Monitoring and evaluation plan for the OH system		1.86
	48_2	Sharing of statistical analysis techniques		1.81
	48_4	Common indicators used to analyze data		1.71
	47_11	Interoperability		1.51

WOAH = World Organisation for Animal Health; IHR = International Health Regulations; INFOSAN = International Food Safety Authorities Network; OH = One Health.

## Data Availability

All data used in the present study are included in the Supplementary Materials, with no mention of sensitive data origins.

## Supplementary Materials

The following supporting information can be downloaded at: https://www.mdpi.com/article/10.3390/pathogens13080710/s1, Table S1: Raw data of responses from 42 institutions from 26 countries gathered and used for statistical analysis. Countries were replaced by numbers to avoid disclosing sensitive data from questionnaire surveys.
